# Raman Spectroscopy for Urea Breath Test

**DOI:** 10.3390/bios13060609

**Published:** 2023-06-02

**Authors:** Evgeniy Popov, Anton Polishchuk, Anton Kovalev, Vladimir Vitkin

**Affiliations:** Institute of Advanced Data Transfer Systems, ITMO University, Birzhevaya Liniya 14, 199034 Saint Petersburg, Russia; avp@itmo.ru (A.P.); avkovalev@itmo.ru (A.K.); vitkin@itmo.ru (V.V.)

**Keywords:** Raman spectroscopy, *Helicobacter pylori*, exhaled breath, δ^13^C, urea breath test

## Abstract

The urea breath test is a non-invasive diagnostic method for *Helicobacter pylori* infections, which relies on the change in the proportion of ^13^CO_2_ in exhaled air. Nondispersive infrared sensors are commonly used for the urea breath test in laboratory equipment, but Raman spectroscopy demonstrated potential for more accurate measurements. The accuracy of the *Helicobacter pylori* detection via the urea breath test using ^13^CO_2_ as a biomarker is affected by measurement errors, including equipment error and δ^13^C measurement uncertainty. We present a Raman scattering-based gas analyzer capable of δ^13^C measurements in exhaled air. The technical details of the various measurement conditions have been discussed. Standard gas samples were measured. ^12^CO_2_ and ^13^CO_2_ calibration coefficients were determined. The Raman spectrum of the exhaled air was measured and the δ^13^C change (in the process of the urea breath test) was calculated. The total error measured was 6% and does not exceed the limit of 10% that was analytically calculated.

## 1. Introduction

Exhaled human breath is a mixture of alveolar, pulmonary, and oral air, which contains biomarkers that allow for tracking of cellular changes [1]. The heterogeneity of human breath outcomes and associated biomarkers is the result of multiple factors, such as variations and inconsistencies in sampling techniques (both within and between individuals), variations inherent in human physiology, the complex interaction of diverse compounds present in the exhaled air, and interference from concurrent medical conditions [1]. The examination of human breath has a potential application as a screening tool in many medical fields, such as gastric cancer [2,3,4], esophageal cancer [5,6], esophagogastric adenocarcinoma [7], lung cancer, and asthma [8,9]. These topics have been discussed in detail in the research literature [10]. The volume of exhaled air at complete rest is approximately 0.5 L per breath cycle for a typical adult male and 400–500 mL per breath for females, and approximately 5% of its volume fraction consists of CO_2_. The latter allows for determining the presence of *Helicobacter pylori* in the organism.

*Helicobacter pylori* can cause many diseases, including chronic gastritis and a peptic ulcer. It has also been associated with extragastric diseases, including atherosclerotic diseases, hepatobiliary diseases, and lung diseases [11]. Various studies have been conducted in order to investigate different diagnostic approaches, the most utilized of which include the histopathologic examination of biopsies, stool antigen test, urea breath test (UBT), and serological testing [12]. Although endoscopy is commonly used as the initial diagnostic method, non-invasive diagnostic methods such as the urea breath test are more preferred due to their convenience for patients.

The urea breath test (UBT) relies on the change in the proportion of ^12^CO_2_ and ^13^CO_2_ in exhaled air. This change occurs due to the *Helicobacter pylori*’s activity, the result of which is the bacteria converting ^13^C-labeled urea into NH_3_ and ^13^CO_2_ [13]. To develop a high-quality device for the δ^13^С determination in human breath, it is necessary to accurately calibrate the device with standard gas samples beforehand.

There are several techniques available for gas sample pattern analysis, including nondispersive infrared sensors (NDIR), Fourier-transform spectroscopy, mass spectrometry, and Raman spectroscopy [14,15,16]. Numerous publications have compared these techniques against each other and against other diagnostic methods such as antigen tests, serological tests, and histopathologic examinations [14,15,16,17,18,19], but NDIR-based sensors are still the most employed for the UBT in laboratories. The sensitivity, specificity, and accuracy of the NDIR method compared to mass spectroscopy are 87.5%, 100%, and 96.3%, correspondingly [20].

Various sources provide conflicting information regarding the UBT’s sensitivity (ratio of true positive results to all positive results) and specificity (ratio of true negative results to all negative results), ranging from 92% to 99% [21,22,23,24]. The UBT’s accuracy (ratio of correct results to all results) fluctuates between 95.3% and 99% [21,23]. The method’s variability in the accuracy values may be related to sensor component instabilities and instrumental errors.

One of the most powerful methods for measuring the volume fraction of isotopologues in a gaseous sample is Raman spectroscopy [25,26,27,28]. The high accuracy of gas analyzers based on Raman spectrometry makes it possible to more accurately determine the ^12^CO_2_ and ^13^CO_2_ volume fraction changes in breath samples. Several studies have demonstrated the potential of using Raman spectroscopy for the UBT [29,30,31], but further investigation is needed to improve the detection of changes in the ^12^CO_2_ and ^13^CO_2_ volume fraction. In this study, we investigate the accuracy of measurements and the stability of results achieved by using a previously developed Raman gas analyzer [25]. The presented results pave the way to the application of Raman spectroscopy for the UBT, allowing for reducing the number of controversial results, which potentially could simplify the diagnosis.

## 2. Materials and Methods

The Raman gas analyzer used is the same as in our recent works [25,32]. In this study, we specified some extra characteristics of the device to estimate its ability to conduct the UBT for the *Helicobacter pylori* identification.

### 2.1. Gas Analyzer

**Figure 1:**
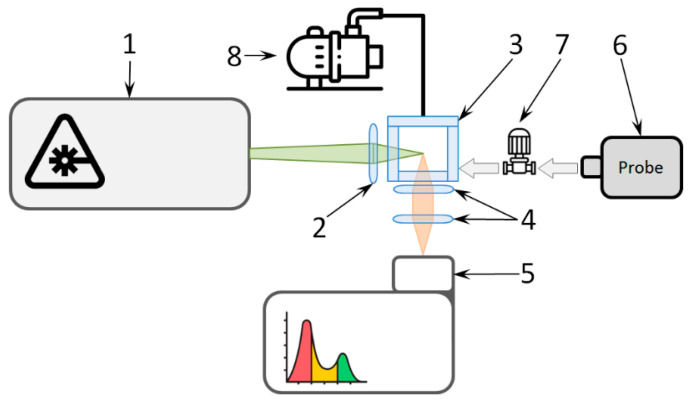
The scheme of the gas analyzer (not in scale): 1—laser; 2—objective; 3—gas cell; 4—f-matcher; 5—spectrometer; 6—specimen; 7—pressure boosting system; and 8—vacuum pump.

The gas analyzer consisted of a gas cell, laser, and high-resolution spectrometer based on the Czerny–Turner scheme with a Hamamatsu HS 101H-2048/250-HR1 CCD camera cooled down to −40 °C. The spectral resolution obtained was 1 cm^−1^ within the spectral region ranging from 200 cm^−1^ to 1500 cm^−1^ (from 540 nm to 600 nm at the laser’s wavelength of 532.1 nm). The gas analyzer scheme is shown in Figure 1.

The root-mean-square deviation (RMSD) of the camera’s dark noise at the photodetector’s minimum temperature did not exceed 12 Analog-to-Digital Converter (ADC) counts (number of digital values that can be assigned to the analog signal, the sensor used has a 6 × 10^4^ count range) in the full image mode with maximum resolution. The quantum efficiency at 25 °C of photodetector temperature was not less than 985 at a 571 nm wavelength. The cooled camera provided lower levels of the dark current and Johnson–Nyquist noise. Working at a temperature of −40 °C increased the signal-to-noise ratio (SNR) up to 100 times compared to room temperature. The influence of temperature on the SNR will be discussed further in more detail.

The laser used was a 5 W, 532.1 nm solid-state laser (MSL-R-532 by CNI Lasers). This laser had a narrow line width up to 0.01 nm to achieve the sample’s spectral resolution of the Raman spectrum of 1 cm^−1^.

In Raman spectroscopy, it is important to use a laser with stable output power and a stable spectrum. We measured these parameters for 5 h and found that the relative deviation from the nominal power for the laser used was 2.1% and the relative deviation of the laser’s central wavelength was 0.001%. The medium value of the laser wavelength was 532,106 nm. The standard deviation of the central laser wavelength position was 0.008 nm, which corresponds to the deviation of one pixel of camera. It can be assumed that the stability of the laser’s central wavelength is higher than the error of the spectrometer used. The 2% deviation of laser power increased the uncertainty of our measurements.

In addition to the gas cell, the gas subsystem included a pressure boosting system and a preliminary cleaning system. The pressure boosting system consisted of a piston-type pump custom designed for this device. The preliminary cleaning system included a vacuum pump.

### 2.2. Samples

Two types of samples were used in this study. The first type consisted of standard gas samples containing ^12^CO_2_ and ^13^CO_2_ in N_2_, with the volume fraction of ^12^CO_2_ ranging from 3.9% to 5.78% and the volume fraction of ^13^CO_2_ ranging from 0.0426% to 0.0647% (provided by the D.I. Mendeleev All-Russian Institute for Metrology; standard sample number 11576-2020). Compounds of samples are shown in Table 1; the δ^13^C value is the difference between the sample and the standard that is calculated by Equation (3).

The second type of samples used in this work was the samples of human breath provided voluntarily by ITMO University employees. The samples were collected in accordance with the generally accepted procedure of conducting the UBT [12]. The samples included two specimens: a base specimen and a diagnostic specimen (taken after ingestion of the 13C-enriched substrate). The specimens were collected into special bags (the volume of the bag was 400 mL) provided with the commercial kit for the UBT (from Isocarb company, Moscow, Russia); the dose of urea was 50 mg [20]. Informed consent was obtained from all subjects involved in this study.

### 2.3. Signal Processing

**Figure 2:**
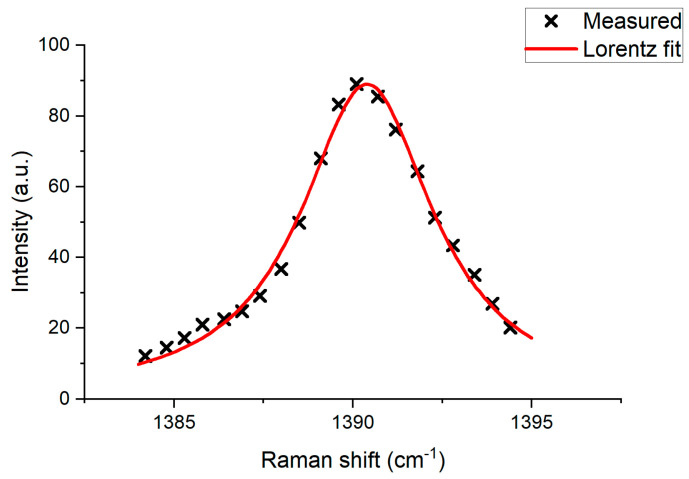
Example of Raman spectrum–Lorentz contour fit: dots—measured values; line—Lorentz function.

The Raman spectrum of a gas sample can be described with a Lorentz function [33]. Figure 2 demonstrates the example of pure ^12^CO_2_ gas Raman spectrum fit with the Lorentz function.

The intensity of Raman scattering $\left(I_{R}\right)$ is described by the following equation [33]:

I_R_ = I_0_·σ_j_·D·dz (1)

where I_0—_the intensity of the laser radiation; σ_j_—the cross section of Raman scattering for the vibration frequency ν_j_ (m^2^·molecule^−1^·moles^−1^); D—the density of molecules; and dz—the length of the optical path.

Equation (1) shows that the amplitude of the registered Raman scattering signal is a linear function of pressure, exposure time, and laser power. To evaluate the amount of the specimen, we normalize the spectral signal amplitude and bring it to a pressure value of 1 atm, laser power of 1 watt, and exposure duration of 1 s. Furthermore, it is essential during normalization to account for the influence of pressure on spectral broadening using a correction coefficient. It is also necessary to consider the dark current, which must also be brought to a zero level.

Equation (1) allows for determining the volume fraction from the analysis of the scattering spectrum. This can be done by using the Raman spectrum peak intensity or area as a basis. In this work, the amount of specimen was determined by the area (S) under the Raman scattering peak of a specific isotopologue (1388 cm^−1^ peak for ^12^CO_2_ and 1370 cm^−1^ peak for ^13^CO_2_). This allowed for a reduction in the thermal noise influence on the stability of the obtained result. To prove this, we conducted the modeling of the CCD camera noise level influence on the volume fraction relative measurement standard deviation using the peak intensity and the area under the peak as a basis for the volume fraction estimation. For the modelling, a Lorentzian profile was assumed, and the CCD noise amplitude standard deviation was defined by the signal-to-noise ratio.

**Figure 3:**
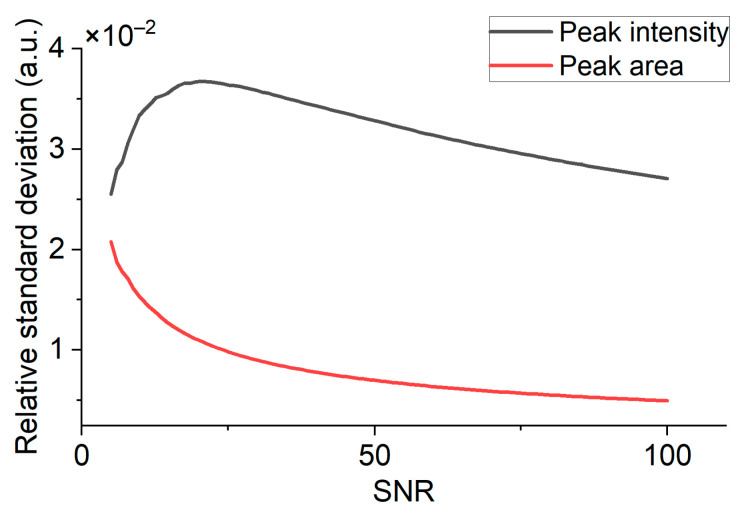
Influence of SNR on a relative standard deviation of volume fraction for two methods: via intensity calculation (blue line) and via the area measurement calculation (orange line).

As seen in Figure 3, the usage of the area under the component’s Raman scattering spectral peak can decrease the relative standard deviation of the measurement about 3 times for a SNR of more than 60. The close values obtained by two methods at a SNR less than 10 indicates a low amount of information obtained under conditions of a low SNR.

The calculation of the volume fraction was performed from signal through calibration functions:

X^12^_C_ = k_12_·S^12^_C_·C_Δw_/(P·W·t) X^13^_C_ = k_13_·S^13^_C_·C_Δw_/(P·W·t) (2)

where k_12_ (k_13_) are calibration coefficients for ^12^CO_2_ (^13^CO_2_) (k_12_ = 0.0123, k_13_ = 7.4·10^−3^); S^12^_C_ (S^13^_C_) is the area under the ^12^CO_2_ (^13^CO_2_) Raman scattering peak; P—the pressure of the specimen in the cuvette; W—laser power; t—exposure time; C_Δw_—the correction coefficient for spectral broadening calculated by Equation (7) (see below).

The calibration coefficients were obtained through the analysis of data from Raman scattering spectra of the standard samples listed in Table 1. Measurements were conducted in a series of 100 measurements for each sample at pressures ranging from 1 to 10 atm and exposure durations ranging from 1 to 600 s. The power of the laser radiation did not vary due to the technical limitations and stayed at 5 ± 0.11 W. The actual power value was recorded at the time of measurement and used for the calibration. As a result, a total of over 7000 measurements were performed. The coefficient of determination for the calibration curve, formed from the results of the measurements, was not less than 0.98.

The volume fractions obtained by Equation (2) are used to calculate the δ^13^C value according to the following equation:

(3) δ13C=X13CX12CsampleX13CX12Cstandard−1•1000‰

where X^13^_C_—the volume fraction of ^13^CO_2_; X^12^_C_—the volume fraction of ^12^CO_2_. The standard value of (X^13^_C_/X^12^_C_) was 0.0112372 for the Pee Dee Belemnite (V_PDB_) [34].

It is important to notice that the volume fraction of ^12^CO_2_ did not change significantly for a certain person during multiple measurements under the same measurement conditions. Therefore, the δ^13^C value could be estimated via ^13^CO_2_ volume fraction difference in diagnostic and base samples:

(4) $$\delta^{13}C=(V_{\mathrm{PDB}}/1000‰)\cdot (1/{X^{12}}_{C})\cdot (X_{13_{C}}^{\mathrm{diag}}-X_{13_{C}}^{\mathrm{base}})$$

where X^13^_C_—the volume fraction of ^13^CO_2_, the upper index is for the base and diagnostic specimen; X^12^_C_—the volume fraction of ^12^CO_2_.

### 2.4. Absolute Uncertainty of Measurement Requirements

The measurement of the exhaled air using the UBT method requires a certain level of absolute measurement uncertainty, which characterizes the variability of the measured value. The volume fraction of CO_2_ in exhaled air typically varies from 4% to 6% [35,36], while the natural abundance of ^12^CO_2_ and ^13^CO_2_ is 98.85–99.04% and 0.96–1.15%, respectively [37,38,39]. While it is important to measure the relative volume fraction of both the ^12^CO_2_ and ^13^CO_2_ components during the UBT, it could be difficult to measure their signals simultaneously due to the differences in the signal strength. Since the change in the ^13^CO_2_ volume fraction is more important for the UBT, the required level of measurement uncertainty was estimated for a 3.8% to 6.3% CO_2_ volume fraction (1.05 safety factor) in exhaled air and a change in δ13C from 3‰ to 100‰. The determination of the required maximum absolute measurement error was conducted according to the following formula:

Δ_abs_ = Δ_rel_·(V_PDB_·[δ^13^C_change_ + 1]·X_CO2_·[1 + (V_PDB_·[δ^13^C_change_ + 1])]) (5)

where Δ_abs_—the absolute error of measurement; Δ_rel_—the relative error of measurement; V_PDB_—the relative volume fraction of ^12^CO_2_ to ^13^CO_2_ in Pee Dee Belemnite (constant at δ^13^C = 0, V_PDB_ = 0.01123720, according to the International Atomic Energy Agency); X_CO2_–the volume fraction of CO_2_ in the exhaled air; and δ^13^C_change_—the change in δ^13^C between the base specimen and a diagnostic specimen (δ^13^C_change_ = [δ^13^C_diag_ − δ^13^C_base_ ]/1000).

**Figure 4:**
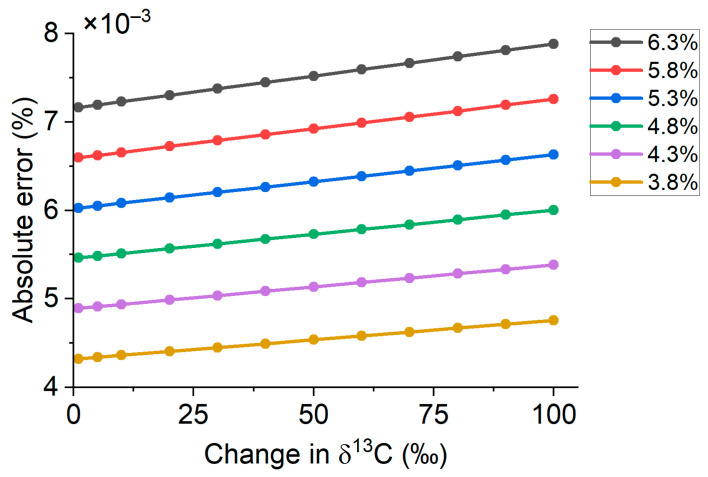
Correspondence of absolute error of the ^13^CO_2_ volume fraction measurement from change in δ^13^C and the CO_2_ volume fraction in exhaled air.

Figure 4 shows the results of the modeling.

For accurate determination of the volume fraction, it is necessary that its measuring resolution does not exceed two standard deviations for a series of measurements. In this case, there will be a 95% probability that the data obtained using this instrument will represent truly different values. The estimated measurement uncertainty value of the ^13^CO_2_ volume fraction required for the UBT was found to be 45 ppm (10% relative error), while the standard deviation of the measurement should be less than 22.5 ppm for a 95% probability. The error budget is therefore limited to 10%.

## 3. Results

### 3.1. Influence of Temperature on SNR

**Figure 5:**
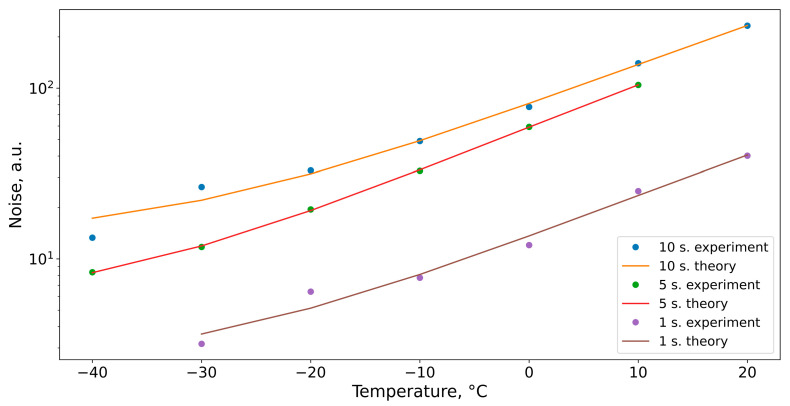
Noise standard deviation as a function of exposure time via different temperatures of CCD camera, points—experimental results, line—fit with Equation (6).

As aforementioned, it is important to estimate the dark current noise and Johnson–Nyquist noise when using a CCD camera. These types of noise are temperature-dependent and affect the results obtained; thus, they should be analyzed to estimate their contribution in the uncertainty of the measurements. During this experiment, Sample 2 (Table 1) was measured at different exposure times and different temperatures of the camera. The SNR was estimated by the N_2_ signal (1.01 × 10^−6^ moles of the 95% N_2_ gas sample, 1 cm^3^ volume, and 0.08 atm pressure). Figure 5 shows the experimental results of the noise standard deviation (STD) measurement at 1, 5, and 10 s of exposure and temperature ranging from −40 °C to 20 °C (note that vertical axes have a logarithmic scale).

It is clearly observed that the noise STD increases with the increase in temperature. The lowest noise was obtained at a camera temperature of −40 °C and exposure time of 1 s. The noise amplitude as a function of temperature was described by the following equation [40] (theoretical curves in Figure 5):

D_e_ = D_e_^0^_diff_·T^3^·exp(Eg/kT) + D_e_^0^_dep_·T^3/2^·exp(Eg/2kT) (6)

where k—the Boltzmann constant; T—the temperature of the sensor; D_e_^0^_diff_—the amplitude of the dark current diffusion noise; D_e_^0^_dep_—the amplitude of the dark current depletion noise; and E_g_—energy of a band gap that varies from temperature E_g_ = f(T) [40].

The determination coefficient (R^2^) was used to estimate the accuracy of approximation. The closer it is to 1, the better fit of data is achieved. The determination coefficient (R^2^) was 0.92, 0.99, and 0.9 at 1, 5, and 10 s of exposure time, correspondingly.

**Figure 6:**
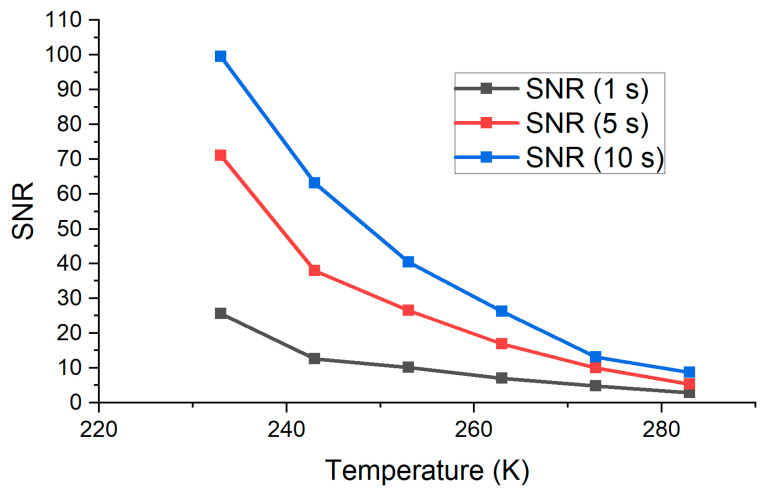
SNR as a function of exposure time via different temperatures of CCD camera.

The Raman scattering intensity of N_2_ can be described with Equation (1). Sample 2 (Table 1) and measured spectra of Raman scattering at different exposure times and different temperatures of the camera were used to estimate the SNR, as shown in Figure 6.

It is clear that the increase in exposure time influences both the noise level and intensity of the Raman spectrum registered by the camera, but for exposure time ranging from 1 to 300 s, the exponential form of SNR dependence on the temperature was observed as the noise level grew more slowly with the exposure time, compared to the signal level. The biggest SNR achieved in this experiment was 200 at −40 °C at an exposure time of 300 s.

All things considered, the dark current noise error was less than 1% at a camera temperature of −40 °C while the temperature and exposure time connected errors were below the error budget.

### 3.2. Influence of Pressure on SNR

The pressure of a gas in the cell affects the SNR since the intensity of the Raman scattering is proportional to the density of molecules in the sample, while the density of molecules itself relates to the amount of the sample (in moles).

The influence of pressure on the Raman line width is well known [41], so the broadening coefficient was taken as 140 × 10^−3^ cm^−1^/atm in our estimation.

Sample 1 (Table 1) was used in this experiment to determine the influence of pressure on the SNR. As shown before, we calculate an area under the Raman line as it has less deviation during the measurement compared to the peak intensity deviation. To consider the effect of spectral broadening, we use the correction coefficient (C_Δw_). This coefficient is calculated using the following formula:

C_Δw_ = 1 + Δw·P (7)

where Δw—the broadening coefficient; P—gas pressure in a gas cell.

**Figure 7:**
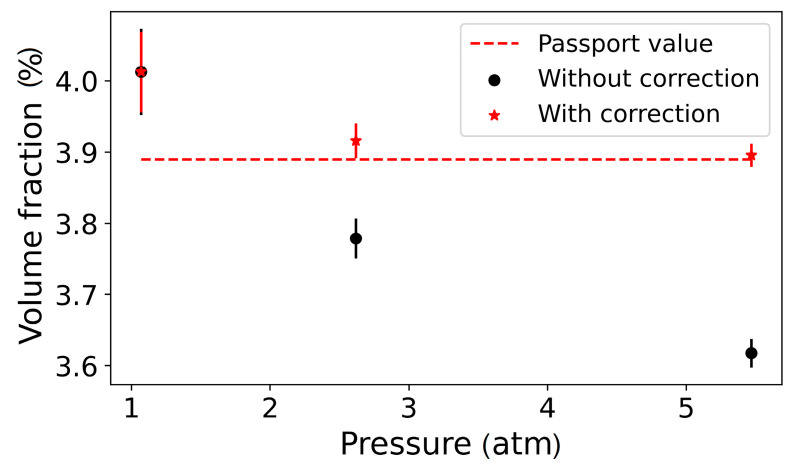
Influence of spectral broadening on signal measured, red stars—with correction of spectral broadening, black dots—without correction of spectral broadening.

Figure 7 shows the resulting influence of pressure on a measured signal. We compared the results of the volume fraction of ^12^CO_2_ measurement obtained for Sample 4 (Table 1) with and without the correction coefficient C_Δw_. The measurements were performed in the range from 1 to 5.5 atm. The known value of the ^12^CO_2_ volume fraction is 3.9%.

As seen from Figure 7, in the case when the correction coefficient was used, the average relative deviation of the mean value from the known value decreased from 5% to 1%. Thus, the correction factor could be accounted to measured data to achieve a more accurate result.

### 3.3. Exhaled Air Measurements

**Figure 8:**
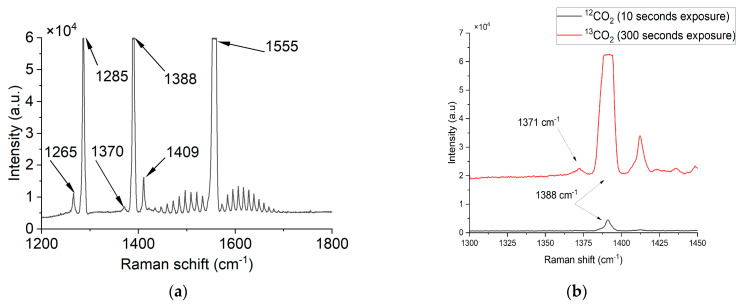
Raman spectrum of the exhaled air: (**a**) the broad range; (**b**) ^12^CO_2_ and ^13^CO_2_ peaks.

The experimentally obtained typical spectrum of the exhaled air Raman scattering at a 60 s exposure in the region from 1200 сm^−1^ to 1750 сm^−1^ is shown in Figure 8a, and the Raman spectrum of ^12^CO_2_ and ^13^CO_2_ at 10 and 300 s exposure times, correspondingly, in Figure 8b.

The exhaled air’s spectrum clearly shows the lines of ^12^CO_2_ (1265 сm^−1^, 1285 сm^−1^, 1388 сm^−1^, and 1409 сm^−1^) and ^13^CO_2_ (1370 сm^−1^), as well as O_2_ (with a characteristic structure of polyads and the most intense peak near 1555 сm^−1^), and is in good agreement with the known data [42,43]. Notably, the ^13^CO_2_ line is significantly lower than the ^12^CO_2_ line due to a lower volume fraction. Due to the fact that in a single sample both ^12^CO_2_ and ^13^CO_2_ were measured at a different exposure time, we can see an overflow of a 1388 cm^−1^ peak of ^12^CO_2_ while the 1371 cm^−1^ peak of ^13^CO_2_ is slightly higher than the noise level.

**Figure 9:**
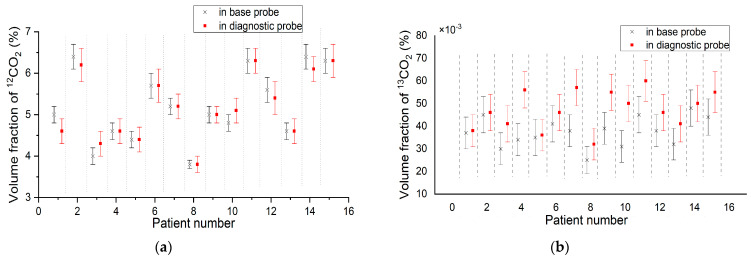
Results of measuring volume fraction in base and diagnostic probes for: (**a**) ^12^CO_2_; (**b**) ^13^CO_2_.

To calculate the ratio of the ^13^CO_2_ to ^12^CO_2_ volume fraction, 10 spectra at 10 s (for ^12^CO_2_) and 10 spectra at 300 s (for ^13^CO_2_) were measured both for base and diagnostic samples. The volume fractions were estimated by the calibration function Equation (2); δ^13^C was then calculated according to Equation (4) for base and diagnostic samples, after which the difference between two samples was calculated. The results of measuring the volume fraction of ^12^CO_2_ and ^13^CO_2_ are shown in Table 2 and Figure 9.

This study involved a group of volunteers (15 persons: 5 males 20–45 years old, 8 females 20–30 years old, and 2 females 50> years old); it included participants who took an antigen test for *Helicobacter pylori* (IgG test) and blood test for *Helicobacter pylori*.

Results shown in Table 2 allow for seeing that the change in the volume fraction of ^13^CO_2_ is close to the standard deviation of the measurement. Table 3 shows that the change in the ^13^CO_2_ volume fraction corresponds to the change in δ^13^C, which is also clearly observed from Equation (4).

The relative measurement uncertainty obtained during this experiment was below 6% while the estimated measurement uncertainty was 4%, which increases the accuracy of volume fraction measurements compared to our previous work [25,29]. The results obtained with the UBT compared with the IgG test are shown in Table 4.

The number of uncertain results achieved with the UBT is 2 against 6 for the IgG test, which decreases the number of extra diagnostics for patients. The percentage of true positive results is 75%, and the percentage of true negative results is 75%.

The results of this experiment show that the obtained values are in good agreement with the typical values measured in other works [13,14,15,16,17,18,19,20,21,22]. Comparing UBT results with IgG, the number of true negative results was 100%. Yet we cannot describe the result as 100% specifically due to the small size of the group and the lack of information about participants. The number of true positive results was 75% (4/7 results) and 24% (3 results) was obtained as an uncertain result. The false-negative results obtained are explained by the inaccuracy during the UBT sampling process (not all participants had not eaten within 6 h prior to sampling). The lesser number of uncertain results was obtained in comparison to the NDIR method, which is an advantage of Raman spectroscopy. Although the sensitivity, specificity, and accuracy should be measured for a larger population to be generalized.

## 4. Discussion

In this work, for the first time to our knowledge, Raman spectroscopy was demonstrated not only as an accurate method for the isotope analysis of human breath, but was also specified as a *Helicobacter pylori* diagnosing instrument in comparison with an IgG diagnostic.

The ^13^CO_2_ volume fraction error had the greatest effect on the obtained results. The measurement uncertainty has included the device measurement error. To reduce this influence, a new quantification method and a pressure-dependent correction factor have been proposed. The method for estimating the amount of a specific component in a sample, based on the area under the Raman spectrum calculation and presented in this work, made it possible to reduce the measurement uncertainty by a factor of three. The evaluation of the spectral broadening influence on the results obtained provided a 40% reduction in the measurement uncertainty due to the introduction of a correction coefficient into the calculation. Using all the methods described above, the device error was up to 5%, resulting in the δ^13^C measurement uncertainty becoming 6%. All results are in good agreement with the results obtained in other works.

The influence of the methodology for making measurements and obtaining samples, as well as the method of calculation, affects the results obtained. Further research is required with more attention to the UBT procedure to assess accuracy and specificity compared to other methods.

## Figures and Tables

**Table 1:** Compounds of standard gas samples.

Sample	^12^CO_2_ Volume Fraction	^13^CO_2_ Volume Fraction	δ^13^C
Sample 1	5.78% ± 0.08%	0.0647% ± 0.0008%	−1.9‰ ± 0.2‰
Sample 2	4.89% ± 0.03%	0.0526% ± 0.0003%	−43.4‰ ± 0.2‰
Sample 3	4.89% ± 0.03%	0.0548% ± 0.0003%	−1.9‰ ± 0.2‰
Sample 4	3.9% ± 0.03%	0.0426% ± 0.0003%	−1.9‰ ± 0.2‰

**Table 2:** Results of exhaled air analysis for participants.

Participant	Volume Fraction of ^12^CO_2_, %	Volume Fraction of ^13^CO_2_ in Base Sample, %	Volume Fraction of ^13^CO_2_ in Diagnostic Sample, %
Participant 1	4.7 ± 0.2	0.037 ± 0.007	0.038 ± 0.007
Participant 2	6.2 ± 0.3	0.045 ± 0.008	0.046 ± 0.008
Participant 3	4.3 ± 0.2	0.030 ± 0.007	0.041 ± 0.008
Participant 4	4.6 ± 0.2	0.034 ± 0.007	0.056 ± 0.008
Participant 5	4.4 ± 0.2	0.035 ± 0.008	0.036 ± 0.007
Participant 6	5.7 ± 0.3	0.041 ± 0.008	0.046 ± 0.008
Participant 7	5.2 ± 0.2	0.038 ± 0.007	0.057 ± 0.008
Participant 8	3.8 ± 0.1	0.025 ± 0.006	0.032 ± 0.007
Participant 9	5.0 ± 0.2	0.039 ± 0.007	0.055 ± 0.008
Participant 10	5.1 ± 0.2	0.031 ± 0.007	0.050 ± 0.008
Participant 11	6.3 ± 0.3	0.045 ± 0.008	0.060 ± 0.009
Participant 12	5.4 ± 0.3	0.038 ± 0.007	0.046 ± 0.008
Participant 13	4.6 ± 0.2	0.032 ± 0.007	0.041 ± 0.008
Participant 14	6.1 ± 0.3	0.048 ± 0.008	0.050 ± 0.008
Participant 15	6.3 ± 0.3	0.044 ± 0.008	0.055 ± 0.009

**Table 3:** Results of δ^13^C change measurement for different participants.

Participant	δ^13^C Difference, ‰	Participant	δ^13^C Difference, ‰
Participant 1	0.26 ± 0.02	Participant 9	5.0 ± 0.2
Participant 2	0.76 ± 0.02	Participant 10	5.6 ± 0.3
Participant 3	3.8 ± 0.2	Participant 11	2.4 ± 0.05
Participant 4	7.4 ± 0.2	Participant 12	2.3 ± 0.05
Participant 5	0.57 ± 0.02	Participant 13	3.2 ± 0.05
Participant 6	1.3 ± 0.05	Participant 14	0.6 ± 0.03
Participant 7	5.6 ± 0.3	Participant 15	2.5 ± 0.05
Participant 8	2.6 ± 0.1		

**Table 4:** Comparison of the IgG and UBT results.

Participant	IgG Result	UBT Result
Participant 1	negative	negative
Participant 2	uncertain	negative
Participant 3	uncertain	uncertain
Participant 4	positive	positive
Participant 5	negative	negative
Participant 6	uncertain	negative
Participant 7	positive	positive
Participant 8	uncertain	negative
Participant 9	positive	positive
Participant 10	negative	positive
Participant 11	negative	negative
Participant 12	uncertain	negative
Participant 13	uncertain	uncertain
Participant 14	negative	negative
Participant 15	positive	negative

## Data Availability

The data supporting the research can be provided by a correspondence author upon reasonable request.
